# Supplementary material for: Multilayered network analysis reveals the centrality of newly discovered Nucleocytoviricota in wastewater treatment plant communities
Source: ISME J. 2026 Oct 21;20(1):wrag267. doi: 10.1093/ismejo/wrag267 (PMC13768754; doi:10.1093/ismejo/wrag267)
Supplement: Supplementary file 1 [file supplementary_material_wrag267.zip › Supplementary_Figures_1.pdf]

Supplementary Figures 1

List of Figures:

Supplementary Fig. 1, page 3. MASH output of average nucleotide identity (ANI) and clustering of the identified *Nucleocytoviricota.* Vertical dashed line indicates >95 % identity.

Supplementary Fig. 2, page 4. Unrooted maximum-likelihood phylogeny of detected *Mriyaviricetes* and sequences from Yutin et al., 2024, based on the major capsid protein.

Supplementary Fig. 3, page 5. Log-log degree distribution of the established interaction network.

Supplementary Fig. 4, page 6. Schematic explanation of the *integration layer*.

Supplementary Fig. 5, page 7. Maximum-likelihood phylogeny of NCVs based on MCP (GVOGm0003). ML tree constructed using IQ-TREE (v2.4.0) and visualized with iTOL (v7.5.1), midpoint rooted. Black circles indicate branch-support (Bootstrap values > 90). Color stripes indicate order assignment, sequences from this study are not assigned. Note that alternative IDs for sequences from this study were used (Supplementary Data, Table S1).

Supplementary Fig. 6, page 8. Maximum-likelihood phylogeny of NCVs based on SFII (GVOGm0013). ML tree constructed using IQ-TREE (v2.4.0) and visualized with iTOL (v7.5.1), midpoint rooted. Black circles indicate branch-support (Bootstrap values > 90). Color stripes indicate order assignment, sequences from this study are not assigned. Note that alternative IDs for sequences from this study were used (Supplementary Data, Table S1).

Supplementary Fig. 7, page 9. Maximum-likelihood phylogeny of NCVs based on RNAPS (GVOGm0022). ML tree constructed using IQ-TREE (v2.4.0) and visualized with iTOL (v7.5.1), midpoint rooted. Black circles indicate branch-support (Bootstrap values > 90). Color stripes indicate order assignment, sequences from this study are not assigned. Note that alternative IDs for sequences from this study were used (Supplementary Data, Table S1).

Supplementary Fig. 8, page 10. Maximum-likelihood phylogeny of NCVs based on RNAPL (GVOGm0023). ML tree constructed using IQ-TREE (v2.4.0) and visualized with iTOL (v7.5.1), midpoint rooted. Black circles indicate branch-support (Bootstrap values > 90). Color stripes indicate order assignment, sequences from this study are not assigned. Note that alternative IDs for sequences from this study were used (Supplementary Data, Table S1).

Supplementary Fig. 9, page 11. Maximum-likelihood phylogeny of NCVs based on PolB (GVOGm0054). ML tree constructed using IQ-TREE (v2.4.0) and visualized with iTOL (v7.5.1), midpoint rooted. Black circles indicate branch-support (Bootstrap values > 90). Color stripes

indicate order assignment, sequences from this study are not assigned. Note that alternative IDs for sequences from this study were used (Supplementary Data, Table S1).

Supplementary Fig. 10, page 12. Maximum-likelihood phylogeny of NCVs based on TFIIB (GVOGm0172). ML tree constructed using IQ-TREE (v2.4.0) and visualized with iTOL (v7.5.1), midpoint rooted. Black circles indicate branch-support (Bootstrap values > 90). Color stripes indicate order assignment, sequences from this study are not assigned. Note that alternative IDs for sequences from this study were used (Supplementary Data, Table S1).

Supplementary Fig. 11, page 13. Maximum-likelihood phylogeny of NCVs based on TopoII (GVOGm0461). ML tree constructed using IQ-TREE (v2.4.0) and visualized with iTOL (v7.5.1), midpoint rooted. Black circles indicate branch-support (Bootstrap values > 90). Color stripes indicate order assignment, sequences from this study are not assigned. Note that alternative IDs for sequences from this study were used (Supplementary Data, Table S1).

Supplementary Fig. 12, page 14. Maximum-likelihood phylogeny of NCVs based on A32 (GVOGm0760). ML tree constructed using IQ-TREE (v2.4.0) and visualized with iTOL (v7.5.1), midpoint rooted. Black circles indicate branch-support (Bootstrap values > 90). Color stripes indicate order assignment, sequences from this study are not assigned. Note that alternative IDs for sequences from this study were used (Supplementary Data, Table S1).

Supplementary Fig. 13, page 15. Maximum-likelihood phylogeny of NCVs based on VLTF3 (GVOGm0890). ML tree constructed using IQ-TREE (v2.4.0) and visualized with iTOL (v7.5.1), midpoint rooted. Black circles indicate branch-support (Bootstrap values > 90). Color stripes indicate order assignment, sequences from this study are not assigned. Note that alternative IDs for sequences from this study were used (Supplementary Data, Table S1).

Supplementary Fig. 14, page 16. Single species collapsed phylogenetic tree of identified NCVs and references, with *Poxvirus fowlpox* (AF198100) as the outgroup and “Yaravirales” removed. For that, a maximum-likelihood tree was calculated for the nine core genes of NCVs (MCP, SFII, RNAPS, RNAPL, PolB, TF2B, Topo2, A32 and VLTF3) using IQ-TREE (v2.4.0) and collapsed with astral. Black circles on the branches indicate branch support >75%.

References

Yutin, N., Mutz, P., Krupovic, M., and Koonin, E. V. (2024). Mriyaviruses: small relatives of giant viruses. *mBio* 15, e01035-24. doi: 10.1128/mbio.01035-24

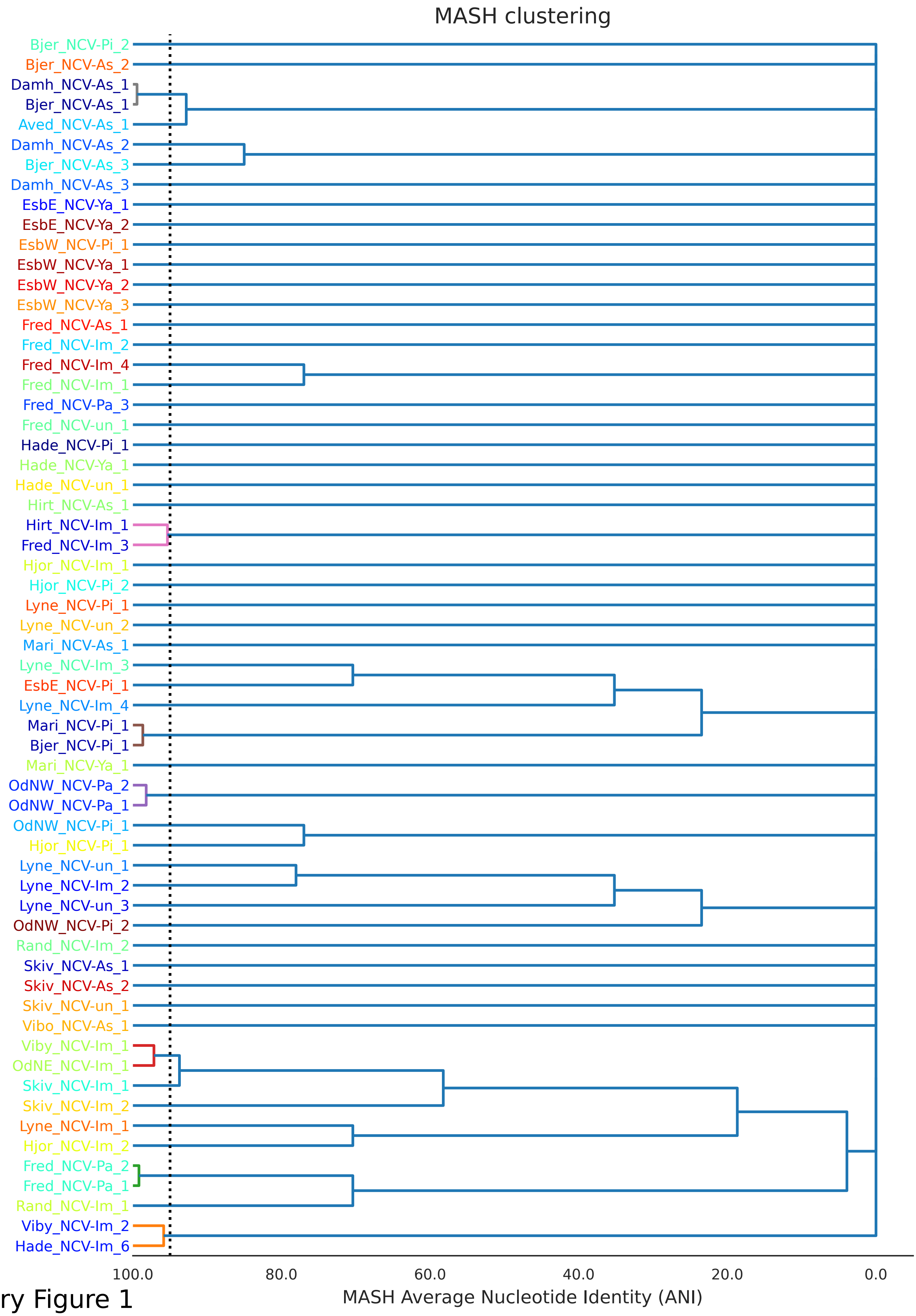

Supplementary Figure 1

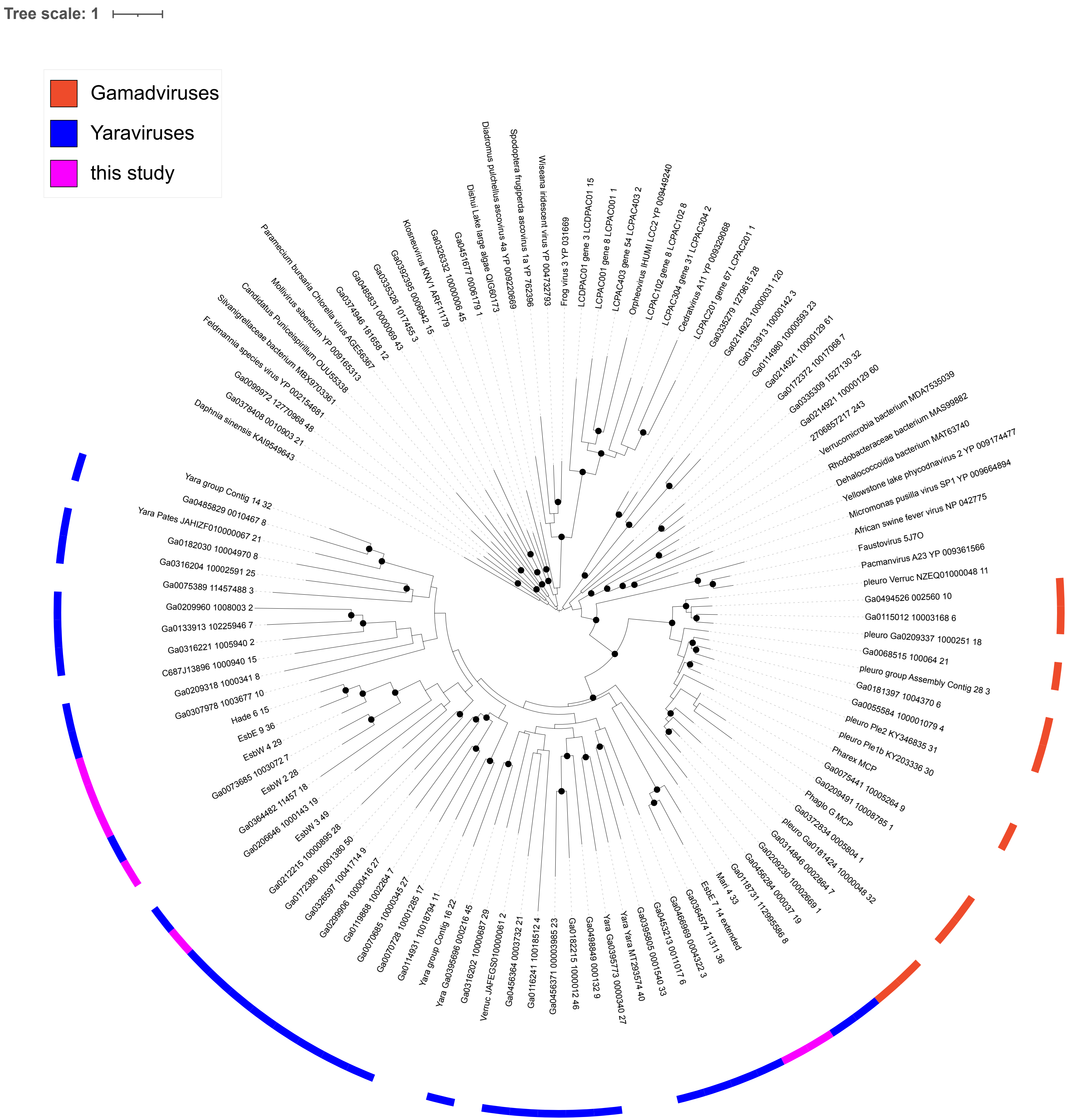

Supplementary Figure 2

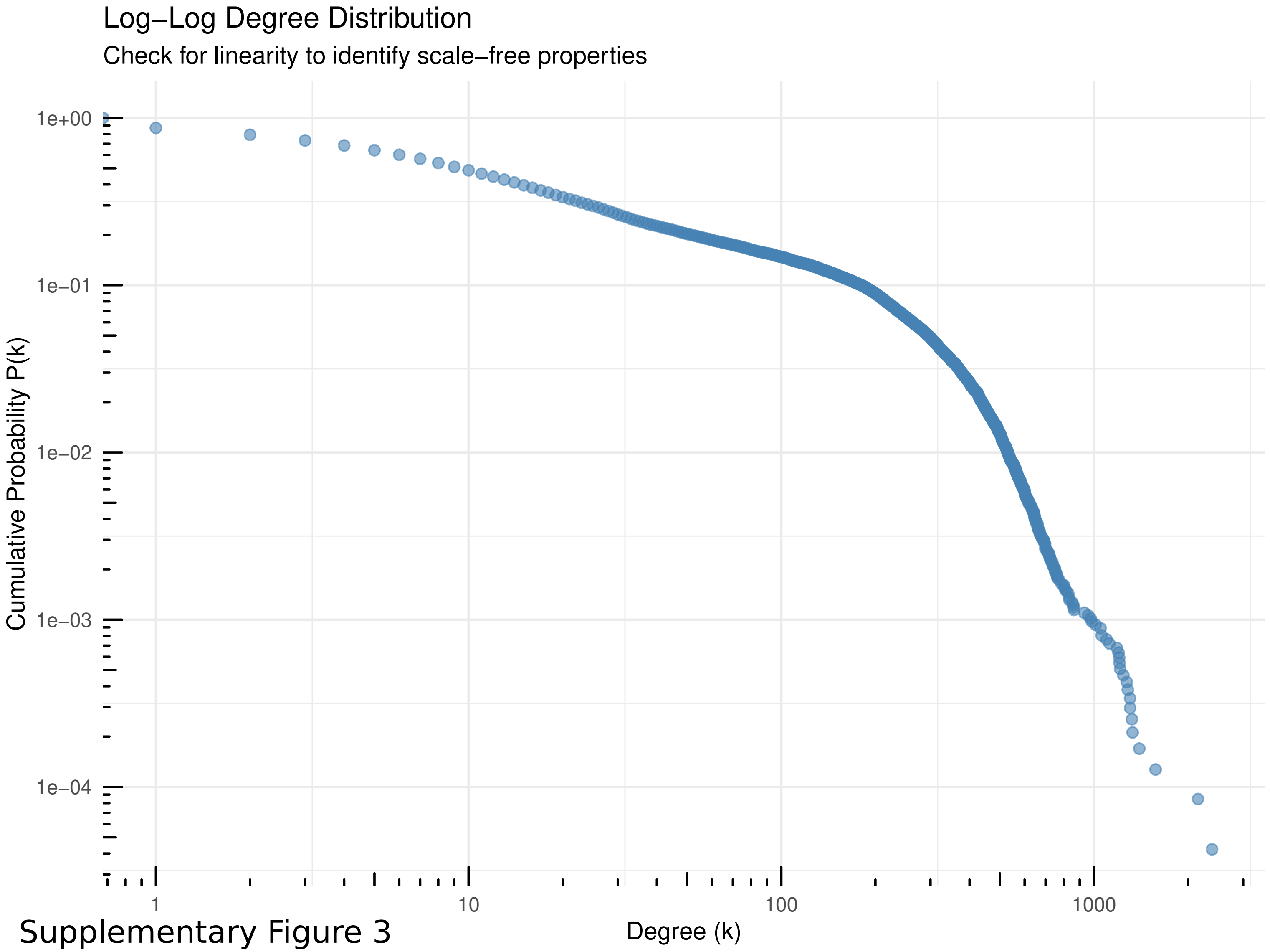

Supplementary Figure 3

Step 1: Mapping long reads to PLV / virophage contigs

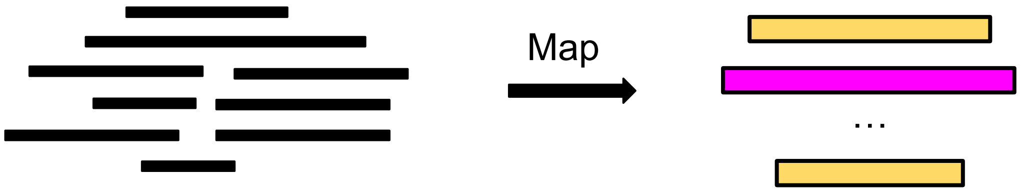

ONT long reads are mapped to 15 + 14 PLV / virophage contigs using minimap2

Step 2: Extraction of “boundary” (b) and “middle” (m) overhangs

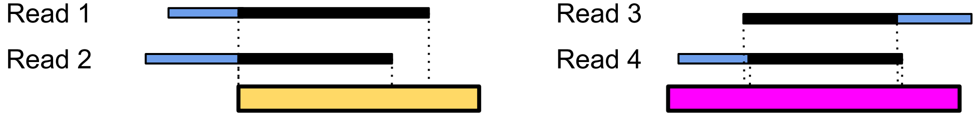

Mapped read overhangs (soft-clipped) were extracted. And overhang was classified as “middle”-overhanging (left hand side example), if the last mapped bp was NOT within 50 bp of the edges of the contigs. Else, an overhang was considered “boundary” overhang (right hand side example).

Step 3: Overhangs were mapped to other contigs (NCVs, MCs)

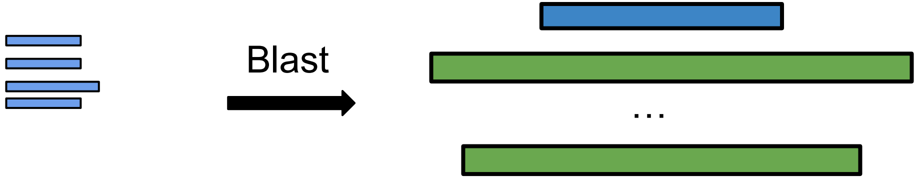

Overhangs were searched against microbial and NCV contigs using blastn (>80 pident, >80qcov).

Further constraints:
Read minimum length: 1000 bp
Minimum mapped read to virophage/PLV: 500 bp
Minimum length overhang: 100 bp

Supplementary Figure 4

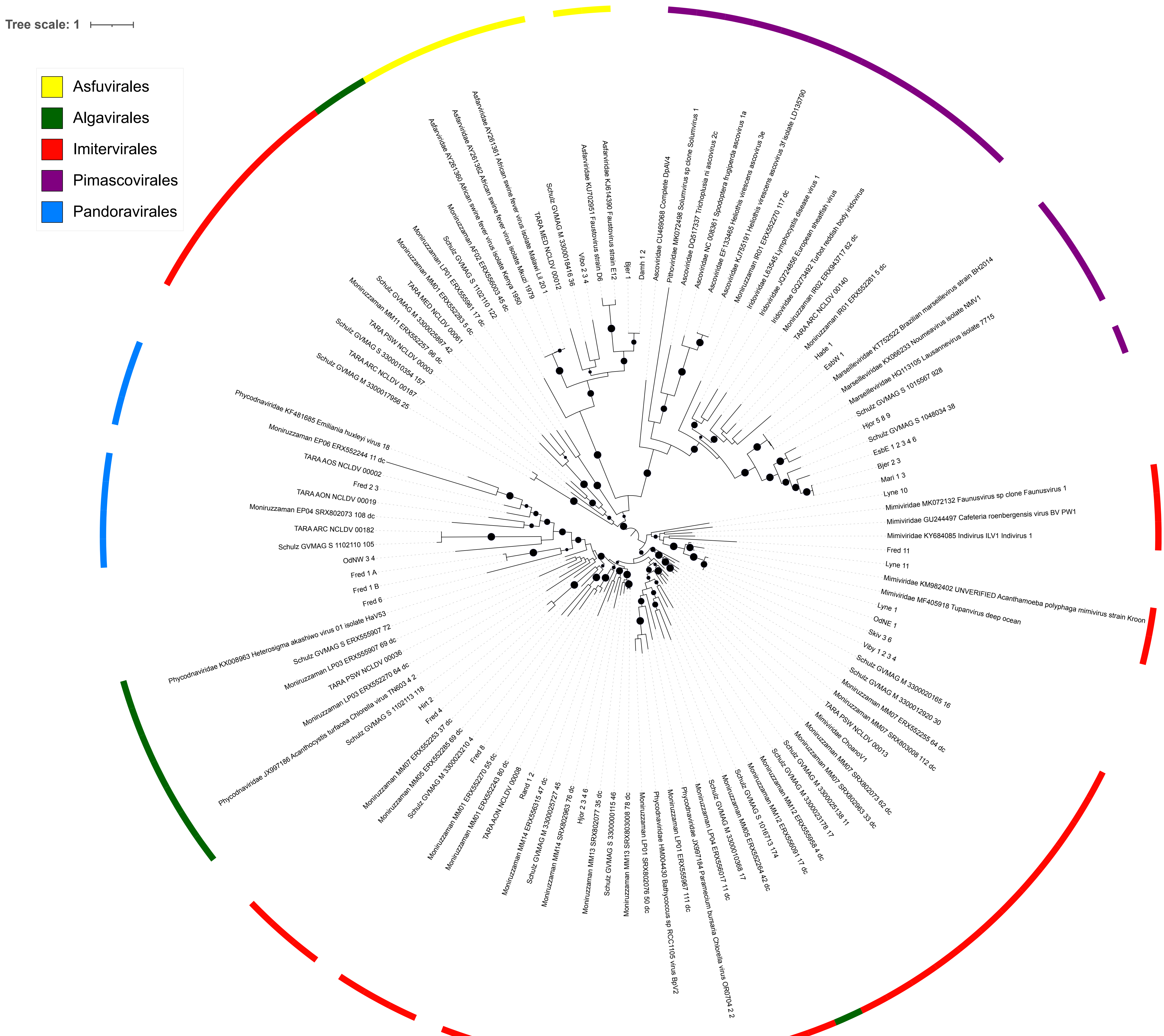

Supplementary Figure 5

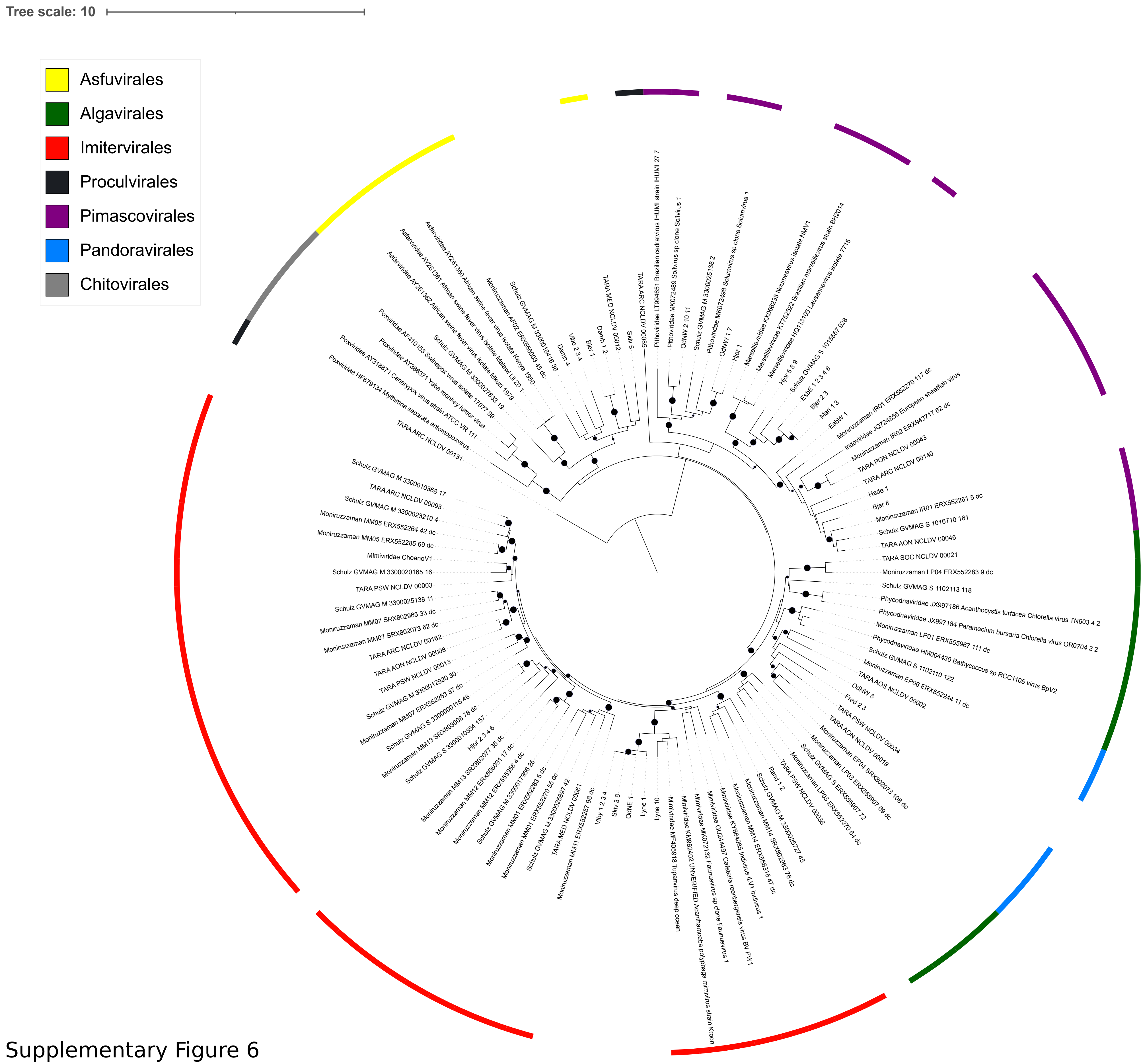

Supplementary Figure 6

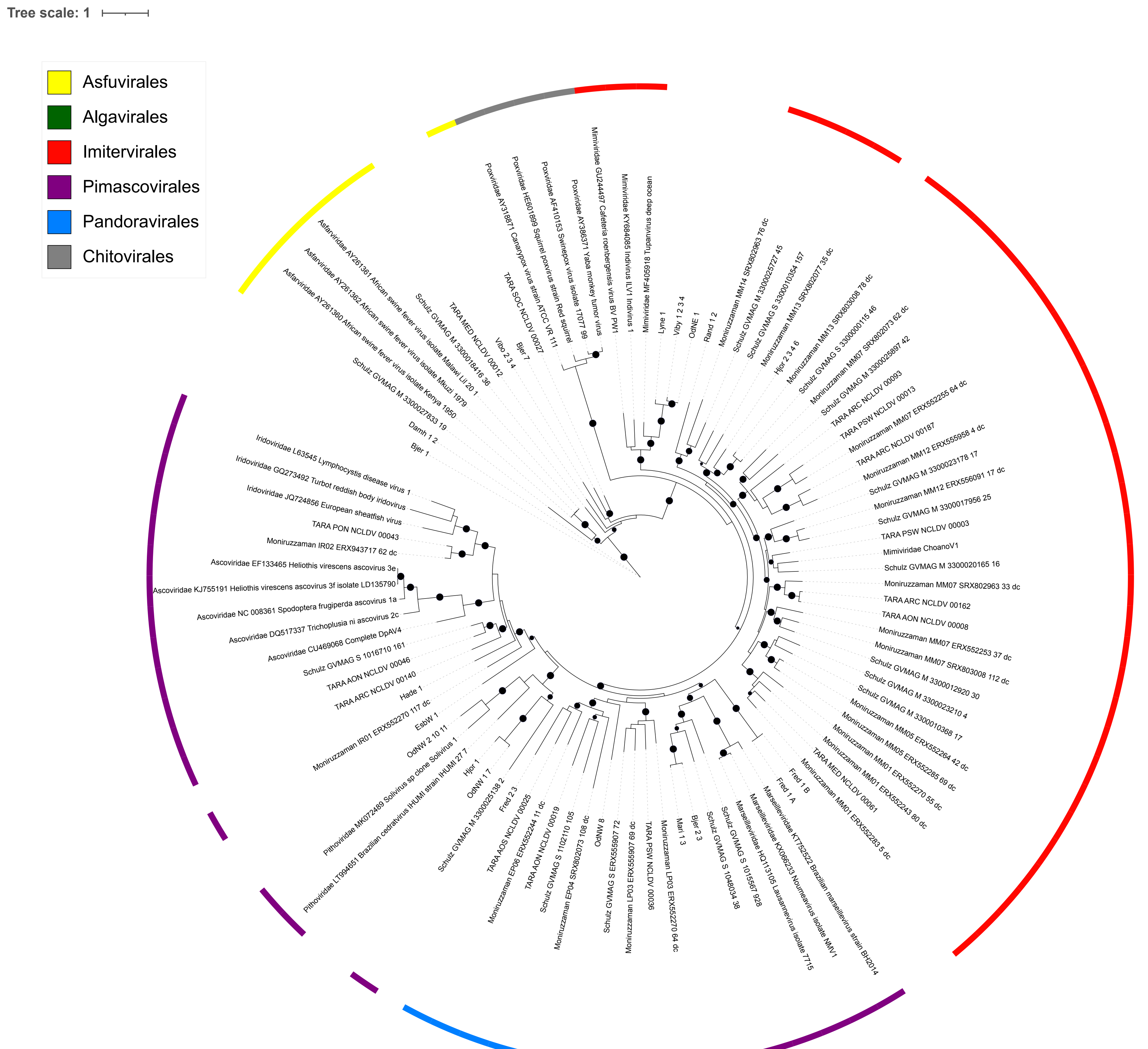

Supplementary Figure 7

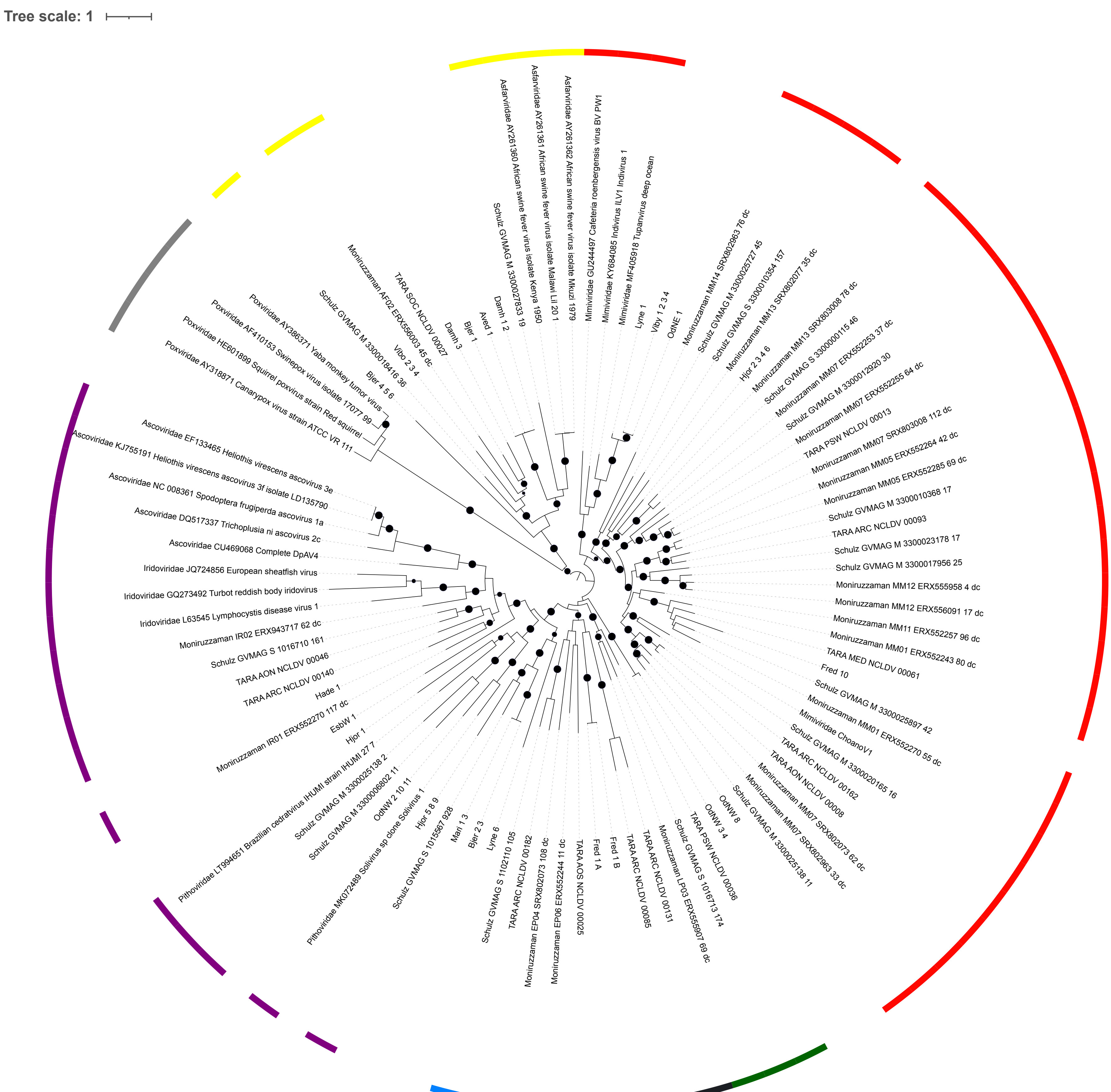

Supplementary Figure 8

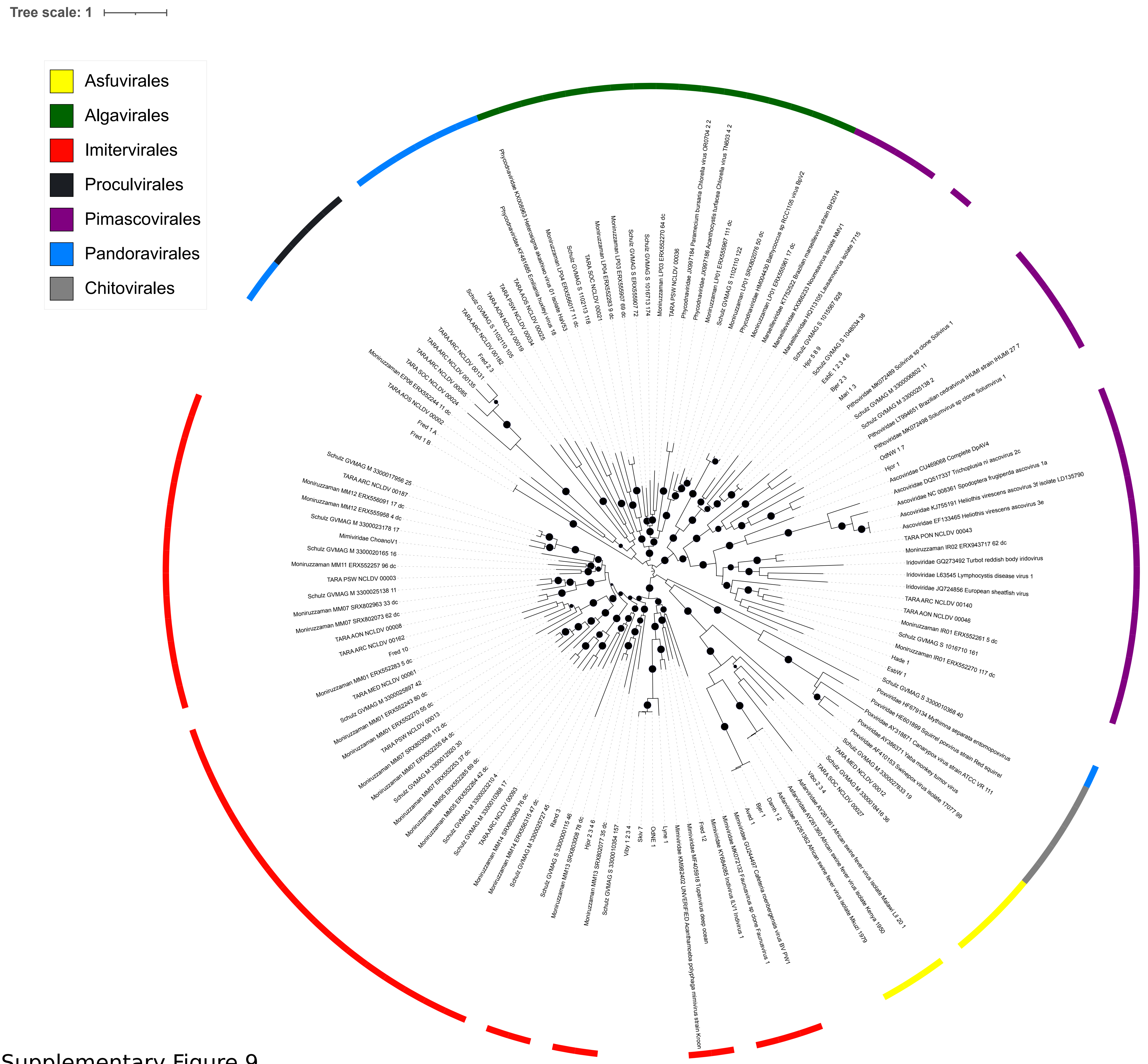

Supplementary Figure 9

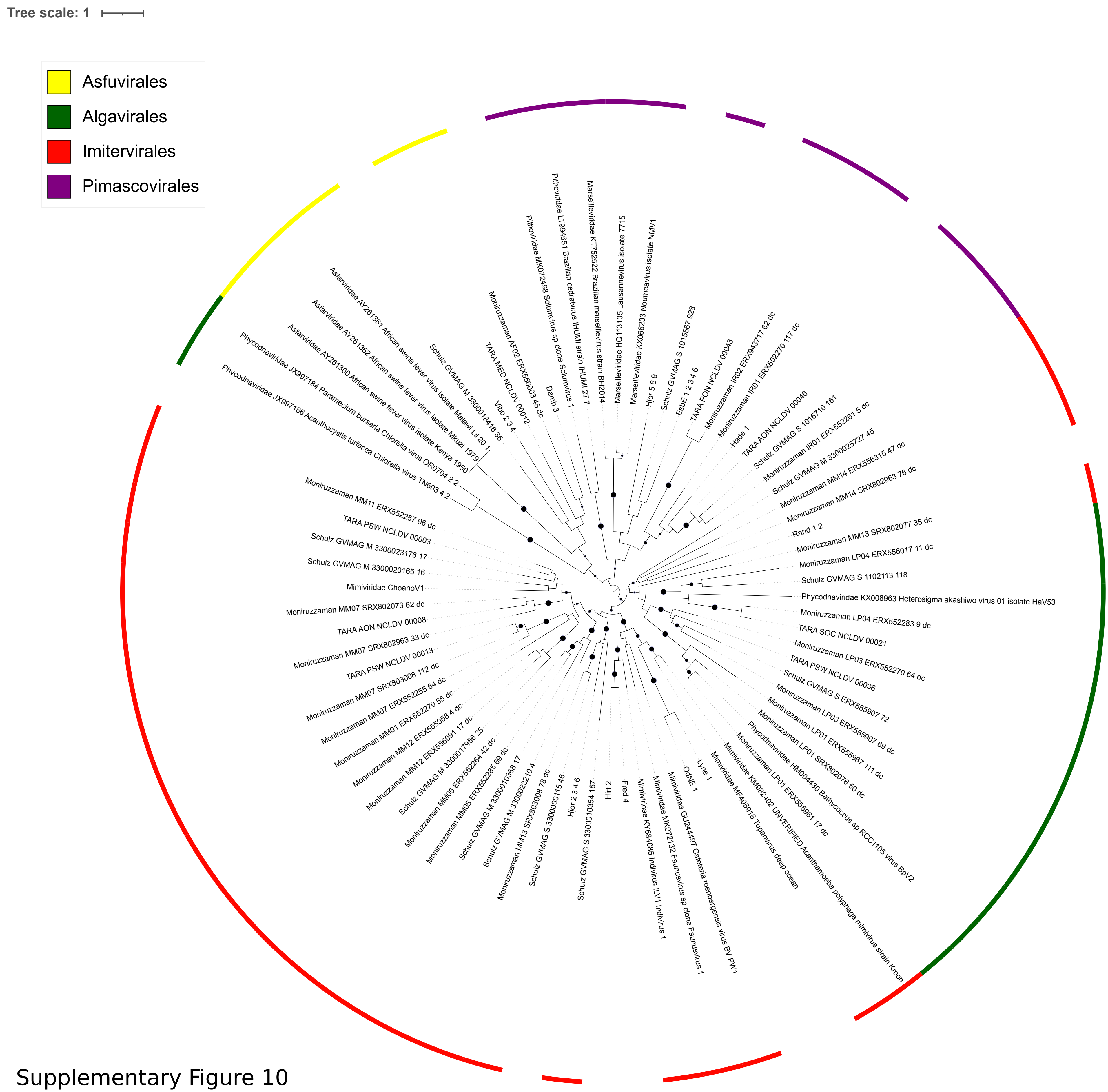

Supplementary Figure 10

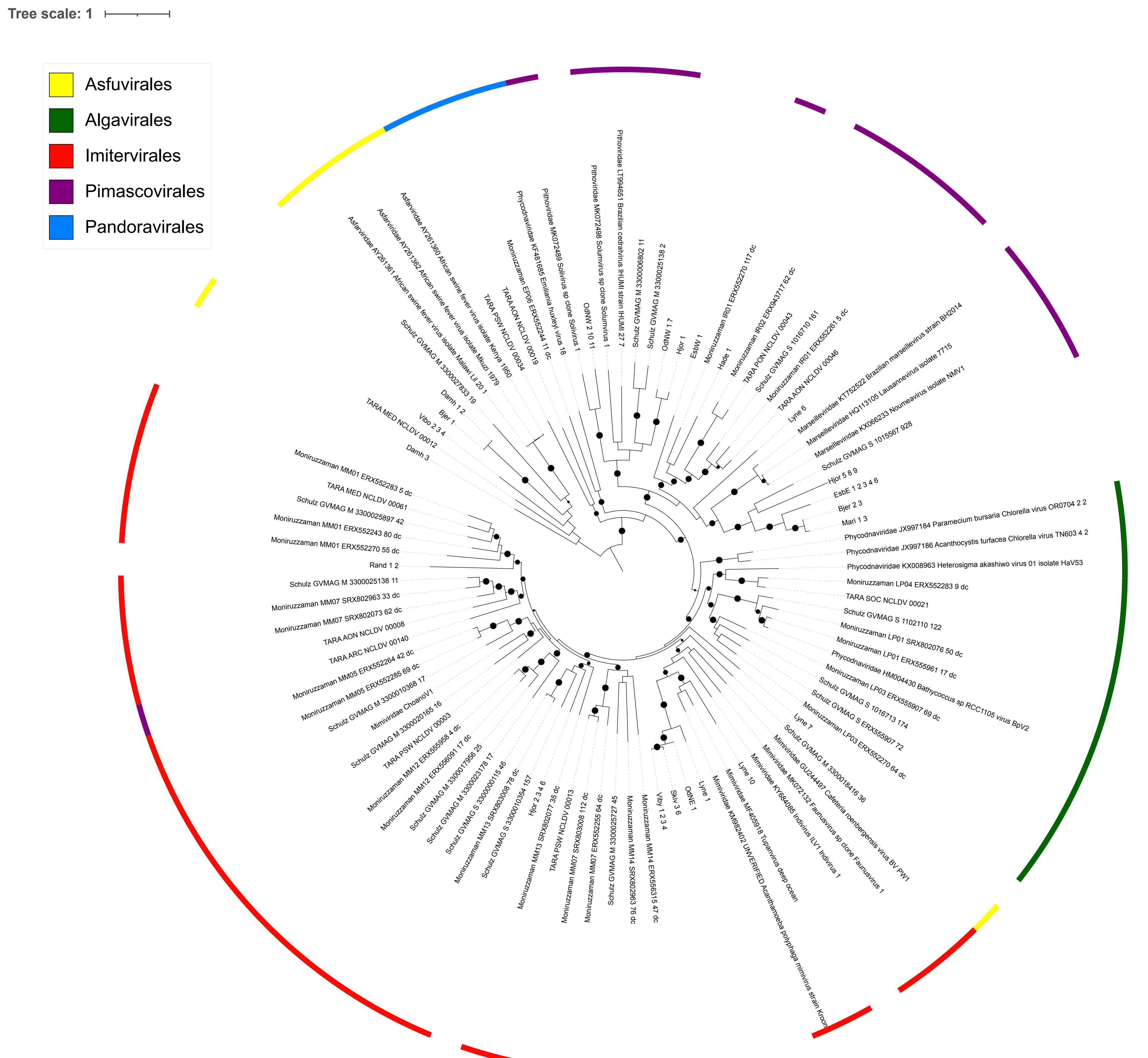

Supplementary Figure 11

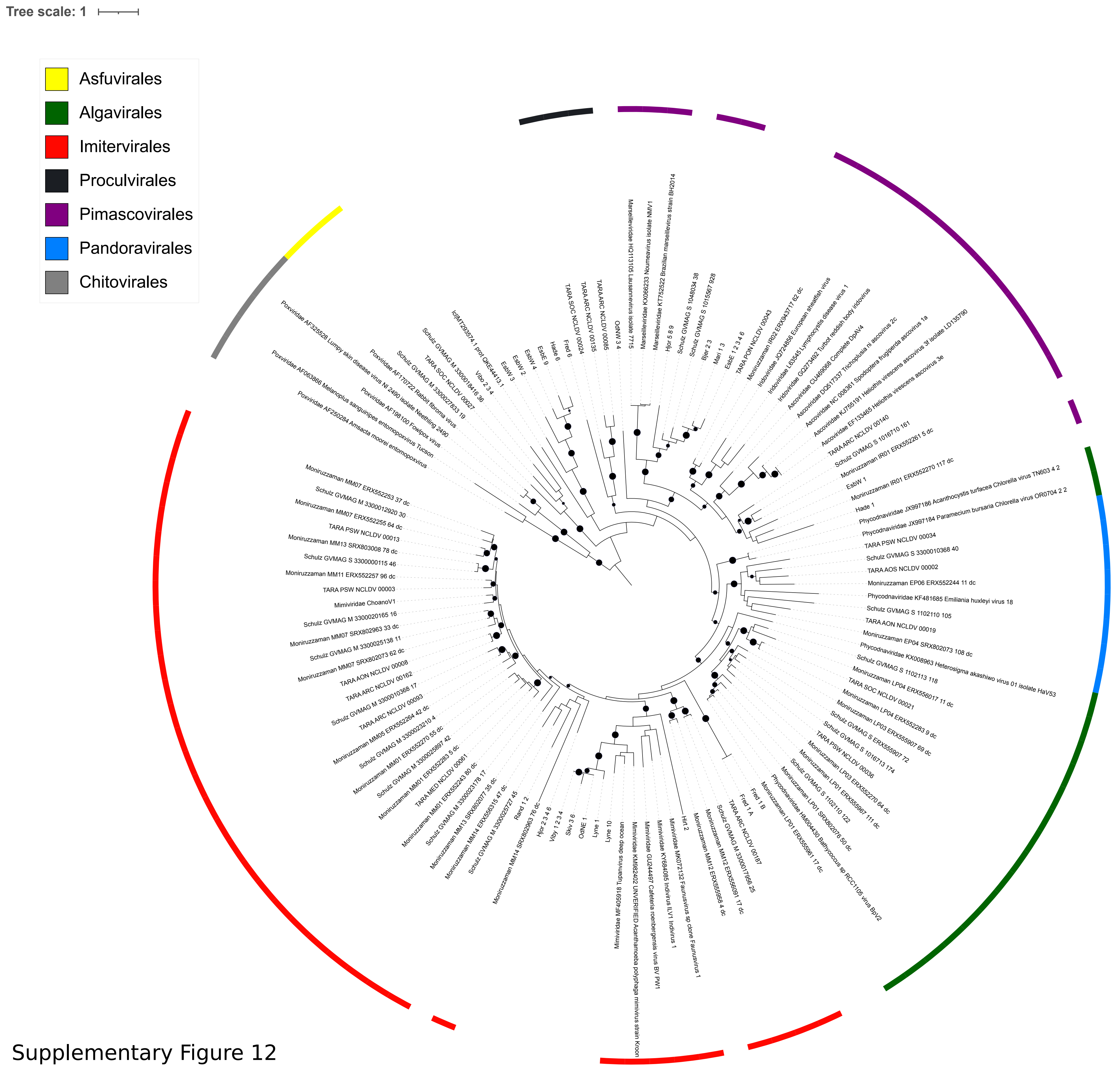

Supplementary Figure 12

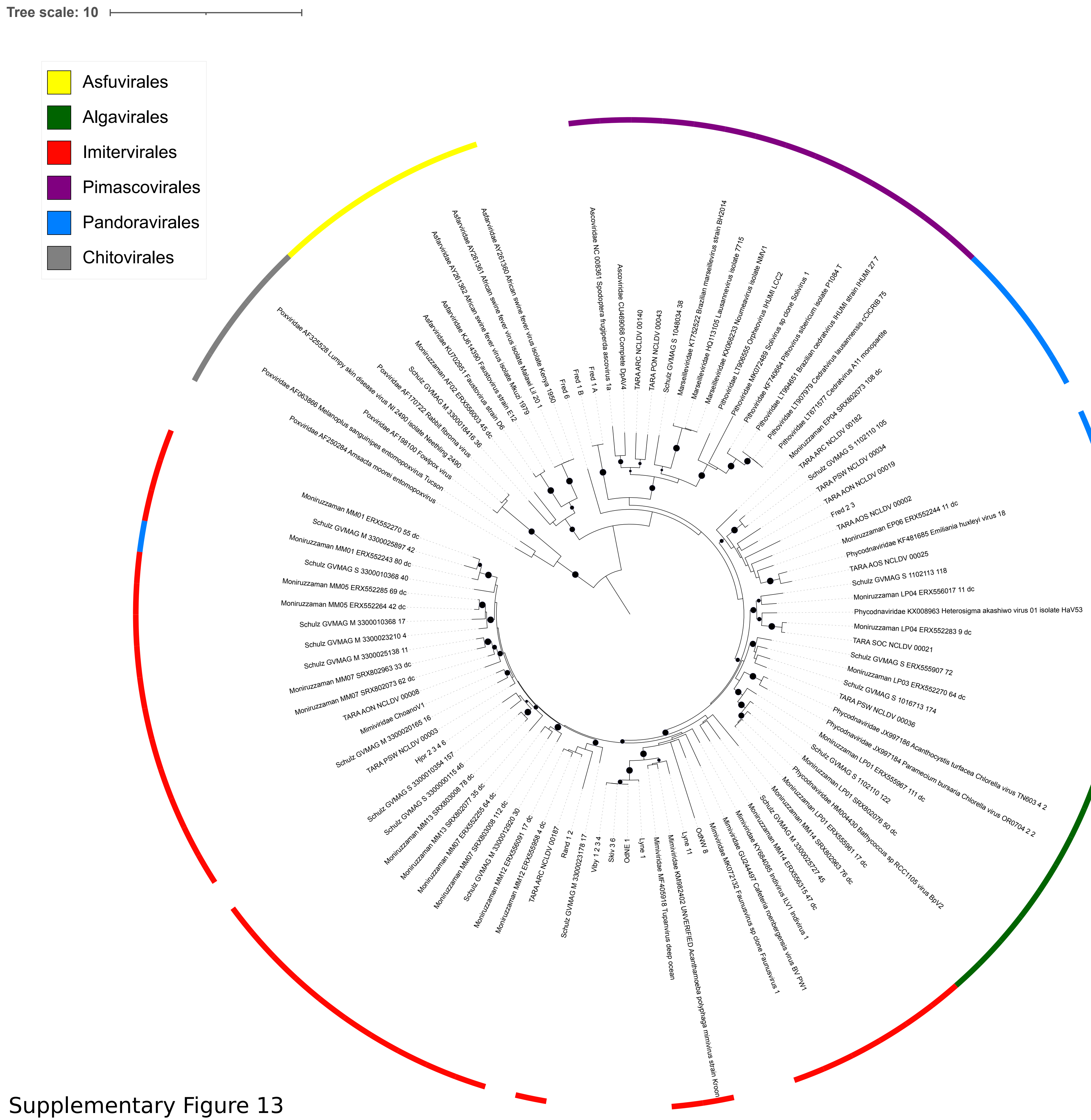

Supplementary Figure 13

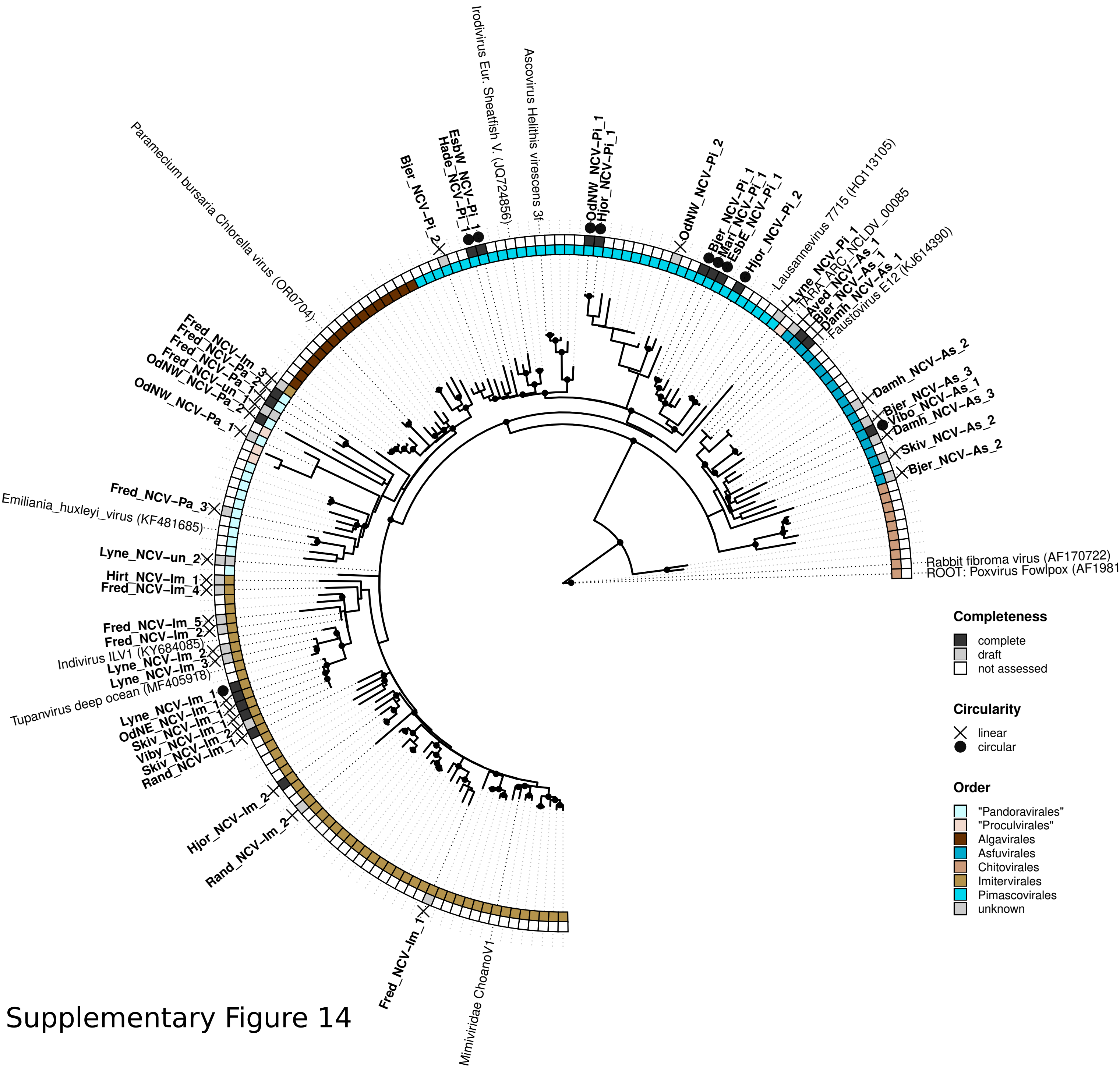

Supplementary Figure 14
