# Supplementary material for: Multilayered network analysis reveals the centrality of newly discovered Nucleocytoviricota in wastewater treatment plant communities
Source: ISME J. 2026 Oct 21;20(1):wrag267. doi: 10.1093/ismejo/wrag267 (PMC13768754; doi:10.1093/ismejo/wrag267)
Supplement: Supplementary file 1 [file supplementary_material_wrag267.zip › Supplementary_material.docx]

# Additional Data

**Supplementary Data 1:** Tabular overview of NCVs (Table S1), custom hmm bitscore cutoffs (Table S2), *Mriyaviricetes* major capsid protein alignment scores (Table S3), virophages (Table S4), PLVs (Table S5), genome accession of publicly available virophages and PLVs used in this study (Table S6). Reference PLV / virophage genomes used in this study for phylogenetics (Table S7).

**Supplementary Figures 1:** ANI and MASH distances of NCVs recovered in this study (Figure S1), maximum-likelihood major-capsid protein trees of detected Mriyaviricetes and sequences from Yutin et al., 2024 (Figure S2), log-log degree distribution of the network (Figure S3), schematic explanation of the integration layer of the network (Figure S4), NCV core gene trees (Figure S5-S13), taxon sampled NCV phylogenetic tree, with “*Yaravirales”* removed (Figure S14).

**Supplementary Figures 2:** Network cluster visualization showing connections among *Nucleocytoviricota, Preplasmiviricota* (Virophages, Polinton-like viruses) and microbial (MC) contigs.
